# Oxidative Damage Induced Telomere Mediated Genomic Instability in Cells from Ataxia Telangiectasia Patients

**DOI:** 10.14293/genint.13.1.003

**Published:** 2022-12-21

**Authors:** Prarthana Srikanth, Amit Roy Chowdhury, Grace Kah Mun Low, Radha Saraswathy, Akira Fujimori, Birendranath Banerjee, Wilner Martinez-Lopez, M. Prakash Hande

**Affiliations:** 1 Department of Physiology, Yong Loo Lin School of Medicine, National University of Singapore, Singapore; 2 Department of Biomedical Sciences, School of Biosciences and Technology, Vellore Institute of Technology, Vellore, India; 3 inDNA Center for Research and Innovation in Molecular Diagnostics, inDNA Life Sciences Private Limited, Bhubaneswar, India; 4 Molecular and Cellular Radiation Biology Group, Department of Charged Particle Therapy Research Institute for Quantum Medical Science Chiba, Japan; 5 Genetics Department and Biodosimetry Services, Instituto de Investigaciones Biológicas Clemente Estable, Montevideo, Uruguay; 6 Associate Unit on Genomic Stability, Faculty of Medicine, University of the Republic (UdelaR), Montevideo, Uruguay; 7 Department of Applied Zoology, Mangalore University, Mangalore, India

**Keywords:** DNA Repair Deficiency, Ataxia Telangiectasia Mutated, Oxidative Damage, Genome Instability, Telomere Dynamics

## Abstract

Our cellular genome is susceptible to cytotoxic lesions which include single strand breaks and double strand breaks among other lesions. Ataxia telangiectasia mutated (ATM) protein was one of the first DNA damage sensor proteins to be discovered as being involved in DNA repair and as well as in telomere maintenance. Telomeres help maintain the stability of our chromosomes by protecting the ends from degradation. Cells from ataxia telangiectasia (AT) patients lack ATM and accumulate chromosomal alterations. AT patients display heightened susceptibility to cancer. In this study, cells from AT patients (called as AT ^-/-^ and AT ^+/-^ cells) were characterized for genome stability status and it was observed that AT ^-/-^ cells show considerable telomere attrition. Furthermore, DNA damage and genomic instability were compared between normal (AT ^+/+^ cells) and AT ^-/-^ cells exhibiting increased frequencies of spontaneous DNA damage and genomic instability markers. Both AT ^-/-^ and AT ^+/-^ cells were sensitive to sodium arsenite (1.5 and 3.0 μg/ml) and ionizing radiation-induced (2 Gy, gamma rays) oxidative stress. Interestingly, telomeric fragments were detected in the comet tails as revealed by comet-fluorescence *in situ* hybridization analysis, suggestive of telomeric instability in AT ^-/-^ cells upon exposure to sodium arsenite or radiation. Besides, there was an increase in the number of chromosome alterations in AT ^-/-^ cells following arsenite treatment or irradiation. In addition, complex chromosome aberrations were detected by multicolor fluorescence *in situ* hybridization in AT ^-/-^ cells in comparison to AT ^+/-^ and normal cells. Telomere attrition and chromosome alterations were detected even at lower doses of sodium arsenite. Peptide nucleic acid – FISH analysis revealed defective chromosome segregation in cells lacking ATM proteins. The data obtained in this study substantiates the role of ATM in telomere stability under oxidative stress.

## Introduction

Genomic stability relies on a wide network of cellular processes, including DNA replication, DNA damage and repair, cell cycle progression, and apoptosis. The DNA damage response (DDR) signaling pathway is regulated by the ataxia telangiectasia mutated (ATM) and ataxia-telangiectasia and Rad3 related (ATR) kinases in response to genomic insult, where ATM protein plays a pivotal role in the DNA double strand break (DSB) damage/repair pathway. ^[ 1, 2]^ The ATM protein (350–370 kDa with 3056 amino acids) is ubiquitously expressed and is distributed into the nucleus. ^[ 3, 4]^ The ATM gene is located on chromosome 11q22.3. ^[ 5]^ Mutations in the ATM gene can lead to the development of ataxia telangiectasia (AT), an autosomal recessive disorder characterized by cerebellar ataxia, oculomotor apraxia, immunodeficiency, choreoathetosis, conjunctival telangiectasias, sensitivity to radiotherapy, and an increased risk of malignancy. ^[ 6, 7]^ Patients with ATM mutations exhibit enhanced telomere attrition and are in a perpetual state of oxidative stress as a result of enhanced telomere shortening. ^[ 8]^ Individuals who are heterozygous for ATM gene mutations have an increased sensitivity to ionizing radiation and are more likely or predisposed to breast, pancreas, and prostate cancers. ^[ 9]^ Actually, ATM gene mutations lead to defects in telomere maintenance in mammalian cells. ^[ 10]^

The conventional role of ATM is in DNA repair with the added responsibility of telomere repair. Previously, acute telomere attrition was observed in peripheral blood lymphocytes from AT patients. ^[ 11]^ In this respect, it was suggested that AT cells are constitutively in a state of oxidative stress, which might explain why enhanced telomere loss with each cell division occurs accompanied by the appearance of chromosome end-to-end fusions and extra chromosomal telomeric fragments. ^[ 8]^ Findings are indicative that ATM may be at the apex in activating defense mechanisms against oxidative stress. Chromosomal stability is primarily maintained by functional telomeres. They are special structures that protect the ends of the chromosomes by capping them. They are nothing but hexanucleotide (TTAGGG) _n_ repeats. The telomeric DNA is associated with numerous proteins and have several critical functions. ^[ 12]^ It has been recorded that activity of ATM kinase is low or minimal in unstressed or normal functioning cells and is chiefly engaged to help the cells to tackle cellular stresses that affect DNA or the chromatin structure. ^[ 13]^

The most lethal DNA lesions are the DSBs as compared to the single stand breaks (SSBs). Over time, cells have developed a variety of responses that can repair DNA damage, preventing cell death. DSBs are repaired by two major pathways, nonhomologous end joining (NHEJ) and homologous recombination (HR). ATM function is primarily required for DSB responses, as demonstrated by AT patients who are extremely sensitive to ionizing radiation. ^[ 14, 15]^ The ATM protein is involved in both pathways. More importantly, a study involving the knockdown of the ATM expression (with an intact BRAC1 gene) resulted in a decrease of NHEJ fidelity, highlighting the importance of the ATM protein for the NHEJ repair. ^[ 16]^

A well-known and potent carcinogenic and genotoxic agent, arsenite is known to cause oxidative damage. It is found in abundance in the Earth’s crust and surfaces through mining and excessive utilization of ground water practices in many parts of the world. ^[ 17]^ Alarming levels of contamination can occur through long-term ingestion of high amounts of arsenite. Heightened incidences of skin, lung, bladder, kidney and liver cancer, and chromosomal anomalies have been related to prolonged exposure to arsenite. Besides, it has been shown that, arsenite induces genotoxic effects in human fibroblasts even at very low concentrations through the induction of DNA strand breaks and the elevation of NADH oxidase activity. ^[ 18]^ It induces oxidative stress by generating reactive oxygen species. ^[ 19]^ We have earlier documented that DNA repair deficient cells are sensitive to genotoxic effects of sodium arsenite as well as other oxidative stress inducing agents. ^[ 19– 24]^

With the aim to understand the role played by the ATM protein in the processing of induced oxidative damage at the telomere region, ATM proficient and deficient (heterozygous and homozygous, respectively) human cells were exposed to two different concentrations of sodium arsenite or 2 Gy of gamma rays and analyzed employing cytomolecular approaches for assessing DNA damage as well as telomere dynamics.

## Materials and Methods

### Cells and culture conditions

AT cells were obtained from Coriell Cell Repositories (Camden, NJ, USA). The human patient fibroblast types were AT ^-/-^ (homozygous knockout strain AG04405A, GM05823E, and GM02052F) and a heterozygous strain AT ^+/-^ (AG 03059A). Normal human lung fibroblasts, IMR-90 cells, also obtained from Coriell Cell Repositories were used as controls in this study. All the cells were maintained consistently in complete minimal essential medium with supplements as suggested by the supplier. All cultured cells were kept in the log phase in a humidified 5% carbon dioxide (CO _2_) incubator at 37°C.

### Treatments

Stock solution of 1 mg/ml sodium arsenite was prepared using double distilled water and diluted with phosphate buffered saline. The cells were treated with arsenite for 24 h. For all the assays, appropriate volumes were added to achieve final concentration of 1.5 μg/ml (11.5 μmol) and 3.0 μg/ml (23 μmol). ^[ 19]^ Every assay had a control without the drug. In addition, another set of same cell types, were irradiated with 2 Gy of ^137^Caesium gamma rays at a dose rate of 1.16 Gy/min (Gammacell ^®^ 40 Exactor, Theratonics, Ottawa, ON, Canada). They were allowed to undergo repair for 24 h and cultured and harvested for chromosomal studies.

### Micronuclei analysis

Cells were incubated with 4.0 μg/ml cytochalasin B (Sigma) in fresh medium for 22 h following treatment with sodium arsenite. The protocol used is based on the method developed by Fenech ^[ 25– 27]^ with modifications. ^[ 28, 29]^ One thousand binucleated cells (BN) with/without the presence of micronuclei (MN) were scored under the Axioplan 2 imaging fluorescence microscope (Carl Zeiss, Oberkochen, Germany) using an appropriate triple band filter.

### Alkaline single cell gel electrophoresis (Comet) assay

Harvested cells were resuspended in Hank’s balanced salt solution (Sigma St Louis, MO, USA), adjusted for cell densities, and mixed with 0.7% low melting point agarose before being applied onto Comet slides (Trevigen, Gaithersburg, MD, USA). The subsequent steps were then carried out in the dark. Following solidification of the agarose at 4°C, slides were subjected to lysis [2.5 M sodium chloride (NaCl)/0.1 M pH 8 ethylenediaminetetraacetic acid (EDTA)/10 mM Tris base/1% Triton-X] at 4°C for 1 h. The slides were then loaded into a gel electrophoresis tank in 0.3 M sodium hydroxide (NaOH)/1 mM EDTA, pH 13, allowed to denature for 40 min, and run at constant 25 V/300 mA for 20 min. Samples were neutralized with 0.5 M Tris-hydrogen chloride (HCl) pH 7.5 for 15 min, dehydrated in a series of ethanol (70%, 90%, and 100%) for 5 min each and then dried at 37°C. DNA was stained with 1:10, 000 SYBR Green (Trevigen) in Tris-EDTA buffer. One hundred randomly selected cells per sample were examined under an Axioplan 2 imaging fluorescence microscope and analyzed using Comet Imager Software (Metasystems, Altlussheim, Germany). The extent of DNA damage was expressed as a measure of comet tail moments, which corresponds to the fraction of DNA in the comet tail multiplied by the tail length.

### Chromosome preparation

After treatment with sodium arsenite (3 μg/ml) or 2 Gy of gamma rays, cells were washed with methoxyethoxymethyl (MEM), and fresh medium was added, followed by incubation for another 24 h without the drug. After incubation, 10 μg/mL KaryoMax Colcemid™ (Gibco, Grand Island, NY, USA) was added to the cells, to arrest them at the metaphase, and then left to incubate for another 9 h. Next, cells were harvested and centrifuged at 1000 rpm for 4 min, followed by supernatant removal. Then, 5 ml of pre-warmed (37°C) 0.075 M potassium chloride (KCl) was added to each sample while vortexing and left to stand at room temperature for 12 min. Subsequently, the cells were centrifuged at 1200 rpm for 5 min, the supernatant was aspirated, followed by two fixation rounds by addition of ice-cold Carnoy’s fixative (acetic acid/methanol, 1:3) while vortexing. Fixed samples were dropped to see if chromosome spreads were present and then stored at 4°C until peptide nucleic acid-fluorescence *in situ* hybridization (PNA-FISH) was conducted. ^[ 22, 30]^

### Peptide nucleic acid-fluorescence *in situ* hybridization

Metaphase spreads prepared from the samples were subjected to two color PNA-FISH using a Cy3 labeled telomere probe and FITC-labeled centromere probes. The procedure for PNA-FISH was described earlier. ^[ 11, 22, 30]^ The chromosomes were counter-stained with 4’6-diamidino 2-phenylindole (DAPI) in Vectashield (Vector Laboratories, Burlingame CA, USA). The Zeiss Axioplan 2 imaging fluorescence microscope (Carl Zeiss) was used to capture 50 metaphases and analyzed for chromosomal breaks and fusions using the *in situ* imaging software Isis (Metasystems). The total number of chromosomes in each metaphase was also recorded. This analysis was performed to determine the nature of chromosomal damage and not for genotoxicity assessment, therefore PNA-FISH was performed to determine the involvement of telomeres in the formation of chromosome alterations.

### Comet-fluorescence *in situ* hybridization assay

The Comet slides (minus SYBR staining) were prepared as explained above in the section “Alkaline single cell gel electrophoresis (Comet) assay”. The Comet-FISH method used here was based on an earlier publication by Santos *et al.* ^[ 31]^ with some modifications for PNA probes. Overnight dehydration of slides in 100% ethanol at 4°C was carried out. This was followed by rehydration of the Comet slide gels for 15 min. Denaturation was carried out chemically by incubating the slides in 0.5 M NaOH/1 M NaCl (heat denaturation was not possible as the agarose would melt). The slides were then subjected to neutralization in 0.5 M Tris-HCl and 1 M NaCl for 15 min. This was immediately followed by dehydration of the gels in an ice-cold ethanol series (70, 90, 100%, 5 min each). Slides were allowed to air-dry. Clean cover slips with hybridization mix were affixed onto dry slides. Care was taken to avoid air bubbles. To facilitate effective hybridization, the slides were placed in a humidified chamber at room temperature for 2 h. This was followed by stringent washing of the slides as described above for PNA-FISH. Gels were again dehydrated in an ice-cold ethanol series (70, 90, 100%, 5 min each). Slides were kept to air dry. Once dry, the slides were counterstained with α-fade SYBR green and placed in a light protected storage box.

### Multicolor fluorescence *in situ* hybridization

Multicolor FISH (mFISH) was performed on metaphase spreads derived from normal and AT cells to detect chromosome abnormalities if any in the samples. mFISH probes (24 Xcyte) were obtained from Metasystems and the slides were subjected mFISH as per the guidelines from the manufacturer as described in Hande *et al.* ^[ 32, 33]^ Metaphase images were captured and analyzed using Isis imaging software (Metasystems) with the Axioplan 2 imaging fluorescence microscope.

### Telomere length measurement by terminal restriction fragment analysis

DNA extraction from cells was performed according to the manufacturer’s protocol using the DNeasy Tissue Kit (Qiagen, Valencia, CA, USA). The telomere restriction fragment (TRF) length analysis assay was performed using the Telo-TAGGG Length Assay Kit (Roche Applied Science, Indianapolis, IN, USA). The Kodak gel imaging system and the Kodak imaging software were used to calculate the quantitative measurements of the mean TRF length. Details were reported earlier. ^[ 34]^

## Results

### Micronuclei frequency increased in a dose dependent manner after arsenite treatment in AT ^-/-^ cells

**Figure 1:**
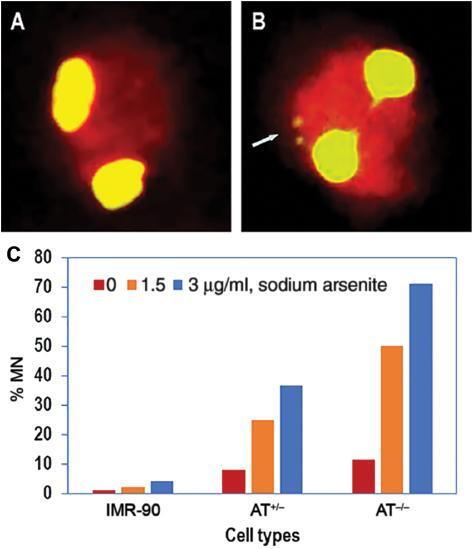
Induction of MN in ATM proficient and deficient fibroblasts following sodium arsenite treatment. Representative fluorescence microscopy image shows a binucleated cell **A)** without micronuclei (MN) and **B)** with MN. The nucleus and cytoplasm are differentially stained by acridine orange in BN cells. **C)** The graph indicates the frequency of MN induction measured in AT ^+/-^ and AT ^-/-^ cells relative to IMR-90 fibroblasts following treatment with sodium arsenite (1.5 and 3 μg/ml). Both AT cell types had increased MN induction at both doses, with increased fold change at the higher dose. One thousand binucleated cells were analyzed for each sample. Abbreviations: ATM, ataxia telangiectasia mutated; BN, binucleated; MN, micronuclei.

A total of 1000 BN cells were scored for each sample. Under untreated conditions, IMR-90 cells (DNA repair proficient cells) showed no MN when compared to AT ^-/-^ cells ( Figure 1A–C). Besides, as shown in Table 1, genomic instability as detected in the form of MN induction is higher in the DNA repair deficient AT ^-/-^ with respect to AT ^+/-^ cells. The number of MN increased with the increase in arsenite concentration, and the extent of genome instability was heightened in AT ^-/-^ cells when compared with AT ^+/-^ or control cells.

**Table 1:** MN frequency induced in human fibroblasts following treatment with sodium arsenite.

	Sodium arsenite (μg/ml)	Total BN scored	BN with respective number of MN				BN with MN	Total MN
			1 MN	2 MN	3 MN	4 or > MN		
IMR-90	0	1000	12	0	0	0	12	12
	1.5	1000	17	2	0	0	19	21
	3	1000	30	4	2	0	36	44
AT ^+/-^	0	1000	52	10	3	0	65	81
	1.5	1000	165	22	6	6	199	251
	3	1000	178	55	16	8	257	368
AT ^-/-^	0	1000	67	15	6	0	88	115
	1.5	1000	170	65	30	28	293	502
	3	1000	202	92	50	44	388	712

Abbreviations: BN, binucleated; MN, micronuclei.

### Increased DNA damage in AT ^-/-^ cells following arsenite treatment was evidenced by single cell gel electrophoresis assay

**Figure 2:**
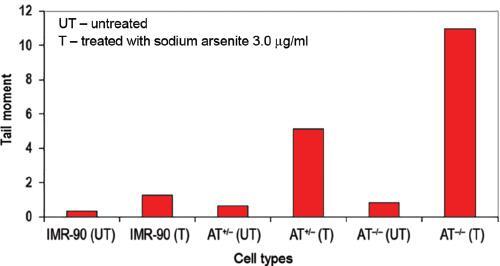
Induction of DNA damage by sodium arsenite in human IMR-90, AT ^+/-^ and AT ^-/-^ human fibroblasts. DNA damage as measured by comet assay following treatment with sodium arsenite. The graph indicates mean values of the tail moments in all cell types A minimum of 100 comets per sample were analyzed.

A sensitive and rapid method of estimating and analyzing low levels of DNA damage at high intensities in single cells is the Comet assay or single cell gel electrophoresis. As many as 50 random cells per slide were captured and analyzed using Comet Imager Software (Metasystems). A significant increase in the tail moment was seen in AT ^-/-^ cells when compared to controls and heterozygous AT knockout cells. On arsenite treatment, a dose-dependent increase in the tail moment of AT ^-/-^ cells was observed. The normal untreated control cells did not show any tail moment or very minimal tail moment ( Figure 2), however, AT ^-/-^ cells treated with a high dose (3.0 μg/ml) exhibited extremely long comet tails as depicted in Figure 2 as tail moment. As presented in Figure 2, AT ^-/-^ cells show significantly greater tail moment than the other cell types. Longer tails were frequently seen in ATM homozygous and heterozygous knockout cells when compared to DNA repair proficient IMR-90 cells.

### Higher telomeric DNA fragments in AT ^-/-^ cells revealed by Comet-fluorescence *in situ* hybridization

**Figure 3:**
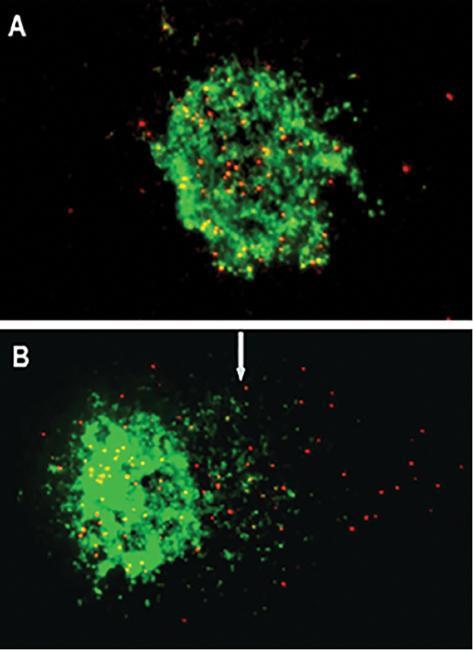
Comet Images of IMR-90 and AT ^-/-^ fibroblasts showing FISH with telomere specific PNA probes. Telomere signals were seen in the comet tail indicating breakage at the telomeric regions. In control cells, minimal distribution of telomere signals beyond the nucleus **(A)**, while in AT ^-/-^ cells treated with sodium arsenite, widespread telomere signals along the comet tail **(B)** representing DNA strand breaks at the telomeres. Abbreviations: FISH, fluorescence *in situ* hybridization; PNA, peptide nucleic acid.

Comet-FISH is a rapid assay suitable for the study of gene-specific and genomic instability or DNA repair in cells and tissues ^[ 35]^ which may predict tumor risk or progression. In this qualitative assay, the DNA in the gel of the Comet slides was processed with telomere specific Cy3-labeled probes, using the PNA-FISH assay. Fifty random cells from each sample were captured and analyzed at the level of single comets. It has been recorded that this method is accurate in revealing telomeric and subtelomeric fragments in comet tails. ^[ 31, 35, 36]^ In comparison to the control cells ( Figure 3A) used in the study, AT ^-/-^ cells showed strong telomere signals in the comet tail ( Figure 3B).

### Chromosome telomere instability in AT ^-/-^ cells as detected by petide nucleic acid-fluorescence *in situ* hybridization

**Figure 4:**
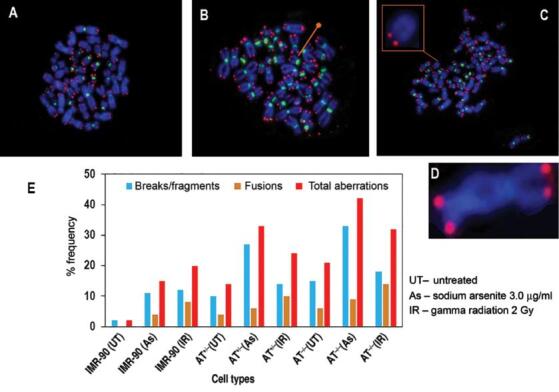
Chromosome aberrations in IMR-90, AT ^+/-^ and AT ^-/-^ cells with or without treatment with sodium arsenite/gamma radiation: PNA FISH analysis. **A**– **D)** Representative images of different types of chromosome alterations detected in metaphases after PNA-FISH with telomere and centromere probes. The red spots represent telomeres stained with Cy3 and the green regions represent the centromeres stained with FITC. **A)** IMR-90 cells with no apparent chromosome alterations. **B)** Chromosomes from untreated AT ^-/-^ cells: red dot showing a chromosome with out p-arm telomeres. **C)** Metaphase chromosome showing loss of telomere signals (zoom box) on chromosomes in AT ^-/-^ cells treated with sodium arsenite. **D)** Chromosome fusions detected in arsenite treated AT ^-/-^ cells. A chromosome with end-to-end fusion is shown. **E)** Percent frequency of different types of chromosome aberrations detected in different cell types following exposure to sodium arsenite or ionizing radiation. Breaks/fragments, fusions and total aberrations are shown in the histogram. Abbreviations: FISH, fluorescence *in situ* hybridization; PNA, peptide nucleic acid.

Metaphase chromosomes were hybridized and processed with telomeric specific Cy3-labeled and centromere specific FITC-labeled PNA probes and chromosomes were counterstained with DAPI ( Figure 4 A–D). A total of 100 metaphase spreads per sample were captured and analyzed. The assay showed numerous aberrations such as fusions (i.e., dicentrics, rings), breaks (acentric fragments), and miscellaneous (such as telomere attrition, extra-chromosomal telomere fragments). It can be seen from Figure 4E that normal cells have a more efficient repair mechanisms than AT ^+/-^ and AT ^-/-^ cells. The total number of aberrations in arsenite-treated AT ^-/-^ cells are significantly higher than heterozygous AT knockout cells and normal fibroblasts. Table 2 shows the aberrations from each sample belonging to untreated, arsenic treated or exposed to ionizing radiation. Chromosome breaks are the most frequent type of aberrations occurring in AT patients. The frequency of breaks is around 1–1.5 times higher than fusions.

**Table 2:** Chromosomal aberrations detected in human lung fibroblasts following treatment with As or ionizing radiation (2 Gy gamma rays).

Cell types	Breaks/fragments*	Fusions	Total aberrations
IMR-90 (untreated)	2	0	2
IMR-90 (As)	11	4	15
IMR-90 (IR)	12	8	20
AT ^+/-^(UT)	10	4	14
AT ^+/-^(As)	27	6	33
AT ^+/-^(IR)	14	10	24
AT ^-/-^(UT)	15	6	21
AT ^-/-^ (As)	33	9	42
AT ^-/-^(IR)	18	14	32

Abbreviations: AS, arsenite; IR, ionizing radiation; UT, untreated.

*Includes number of chromosome fragments without telomere signals and centric fragments. A minimum of 50 metaphases per sample were analyzed.

Upon treatment with arsenite (3.0 μg/ml), AT ^-/-^ cells are more susceptible to damage than control and AT ^+/-^ cells ( Figure 4E, Table 2). On the other hand, it has been observed that arsenite treatment can increase the production of reactive oxygen species, which can induce DNA damage. ^[ 37]^ Similarly, cells from patients with AT homozygous mutation are highly sensitive to radiation when compared to the other samples ( Figure 4E). The total number of chromosomal aberrations recorded for AT ^-/-^ patients under irradiated conditions have shown marked increased over the rest of the samples, as shown in Table 2.

### Chromosome translocations detected in AT ^-/-^ cells uncovered by multicolor fluorescence *in situ* hybridization

**Figure 5:**
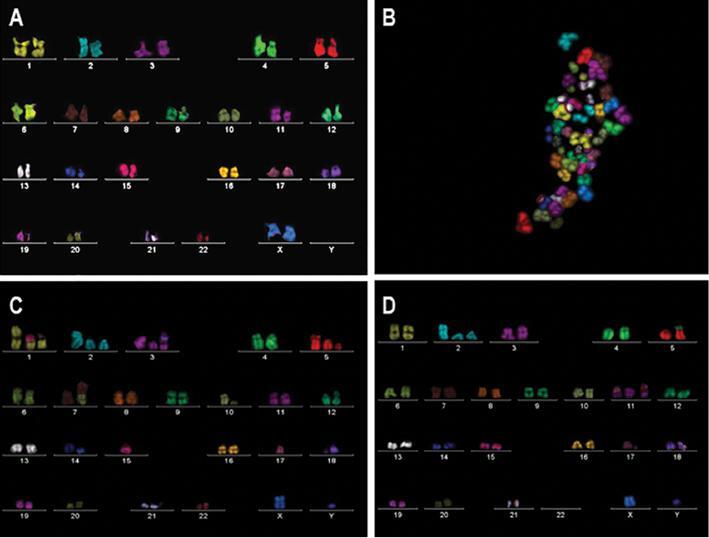
mFISH analysis on metaphase chromosomes from IMR-90, AT ^+/-^ and AT ^-/-^ fibroblasts following treatment with sodium arsenite or gamma radiation. **A)** Normal karyotype of IMR-90 cells. **B)** Arsenite treated metaphase spread AT ^-/-^ cells showing abnormal separation of sister chromatids. **C)** Karyotype of AT ^-/-^ cells following exposure to radiation showing multiple translocations: t(7;10), t(1;15), break(5p), fragment (18). **D)** Karyotype of AT ^-/-^ cells following exposure to radiation showing multiple translocations: der(5), t(5;12), t(11;17), t(11;22). Abbreviation: mFISH, multicolor fluorescence *in situ* hybridization.

All cell types were analyzed using mFISH after treatment conditions (untreated, arsenite treated, and irradiated). In Figure 5A, a karyotype of untreated IMR-90 metaphase spread is displayed while abnormal arsenite treated AT ^-/-^ cells and AT ^-/-^ cells with multiple complex aberrations following irradiation are shown in Figure 5B and Figure 5C–D, respectively. In addition, some spreads showed homologous chromosomes physically together on a single spread, while the majority of the spreads showed chromosomes to be scattered in a spread.

### Measurement of telomere length by telomere restriction fragment length assay

**Figure 6:**
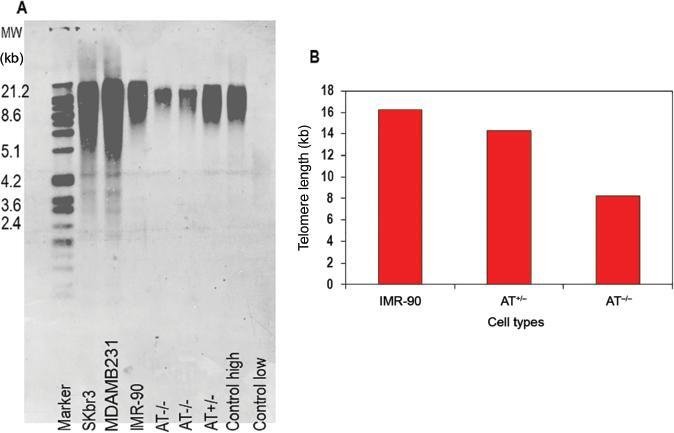
Telomere length estimated by the analysis of the TRF. **A)** Total genomic DNA from different cell types was assessed for telomere length using the TRF assay. Southern blotting of TRF. SKbr3 and MDAMB231 are breast cancer cells to show the difference between fibroblasts and cancer cells. **B)** Telomere length in kb is shown on y-axis for untreated IMR-90, AT ^+/-^ and AT ^-/-^ fibroblasts. Shortened telomeres are observed in AT ^-/-^ cells. Abbreviations: MW, molecular weight; TRF, terminal restriction fragment.

As measured by this assay, it is observed that AT ^-/-^ cells show acute telomere attrition when compared to AT ^+/-^ cells and normal cells even under treated conditions ( Figure 6A–B).

## Discussion

AT patients are known to be sensitive to oxidative damage and ultraviolet (UV) induced DNA damage. ^[ 37– 39]^ Our data clearly show that the repair deficient AT ^-/-^ cells are sensitive to oxidative induced stress. In our study, we have investigated the role of telomere associated chromosomal aberration frequency in ATM heterozygous and homozygous knockout cells in comparison to DNA repair proficient cells. Our results showed hypersensitivity of AT ^-/-^ cells and moderate sensitivity of AT ^+/-^ in response to arsenite and ionizing radiation induced oxidative stress. The data showed significant susceptibility of AT ^+/-^ and AT ^-/-^ cells to arsenite induced oxidative stress and irradiation when compared with the IMR-90 cells as controls. The MN assay is a reliable cytogenetic test that can detect genomic damage to a large extent and also give information on cell survival. ^[ 40]^ The higher the number of MN, the greater the instability and very soon thereafter cells perish. In this respect, we have detected two to three MN in AT ^-/-^ cells ( Figure 1C) when compared to AT ^+/-^ ( Figure 1B) and IMR90 control cells ( Figure 1A). This gives more insights into how ATM homozygous-deficient cells are more unstable and susceptible to mutation and death or apoptosis.

Previous studies reported the role of ATM in DNA repair surveillance and recruiting the repair machinery to the site of DNA damage. ^[ 8, 37, 41, 42]^ We found that AT ^-/-^ cells ( Figure 3B) showed telomere signals in the DNA damaged area qualitatively studied in the comet tails in our Comet-FISH assay. Earlier studies have shown this method to be effective in displaying telomeric and subtelomeric fragments in comet tail. ^[ 36]^

Treatment with arsenite for 24 and 48 h in PARP ^-/-^ cells showed telomere attrition due to oxidative stress. ^[ 19]^ AT ^-/-^ cells have shown a significant increase of genetic damage ( Figures 1C, 2, and 4E) both at low and high concentrations of arsenite treatment, and this could probably be due to the fact that ATM protein is at the apex of recruiting DNA repair machinery once it senses damage, and the lack of it simply endangers cell survival and increases susceptibility to tumorigenesis.

Using PNA-FISH and mFISH, we were able to identify a variety of chromosome alterations induced by arsenite or ionizing radiation. Breaks and fragmentation frequencies of AT cells were significantly higher when compared to the IMR-90 cells employed as controls. Through m-FISH, multiple translocations involving three or more chromosomes were detected frequently in AT ^-/-^ cells ( Figure 5C, D). Our results also show that the arsenite-induced oxidative stress leads to more chromosome fragmentation than fusions or dicentrics formation when compared to ionizing radiation ( Figure 4D). Previous studies have reported that intrachromosomal rearrangements and deletions are produced more efficiently by ionizing radiation than chemical cytotoxic agents. ^[ 33]^ Absence of sufficient telomere bases at chromosome ends in the cells defective in ATM protein can cause the chromosomes to fuse and result in dicentrics and complex chromosomal translocations. On the other hand, it has been demonstrated that oxidative stress reportedly accelerates telomere attrition ^[ 43– 46]^ by inhibiting telomerase and disrupting the recognition by telomere-binding proteins which contributes to telomere uncapping. ^[ 43, 46, 47]^ This substantiates that telomeric DNA may be hypersensitive to oxidative DNA damage.

Cells lacking ATM protein had chromosomal segregation deficiency during mitosis and physical separation of sister chromatids ( Figure 4B), which was observed through the PNA-FISH analysis. This phenomenon might have resulted from higher genomic instability and the apparent lack of functional telomeres in these cells. ^[ 8, 37]^ Genomic instability could be the result of shorter and dysfunctional telomeres at the tails of the AT ^-/-^ and AT ^+/-^ cells. In mFISH, as displayed in Figure 5B, chromosomes from two nuclei where the homologous chromosomes appeared to be adjacent to each other instead of being scattered in the spread (data not shown). The observation reiterates the role of telomeres in homologous pairing, meiotic, and mitotic segregation. Positioning of telomeres within the nucleus is highly specific and dependent on the telomere interactions with the nuclear envelope directly or indirectly (through chromatin interacting proteins). It is possible that telomere chromatin structure might have a regulatory role in telomere movement where ATM may play a role. Moreover, inactivation of ATM has been observed to enhance the frequency of chromosome end association and telomere loss. ^[ 48]^

TRF length analysis showed that the AT ^+/-^ and AT ^-/-^ cells had considerably shorter telomeres when compared to the normal IMR-90 fibroblast cell type owing to the end replication errors experienced by telomeres. ^[ 49]^ In this respect, it has been suggested earlier that after telomeres replicate, the ends must be recognized as DNA damage allowing end replication to occur. ^[ 50]^. DNA damage will elicit a repair response by the HR pathway in which ATM is involved. ^[ 51]^ Due to the absence of ATM in AT ^-/-^ cells, no HR gets recruited and so telomeres do not replicate completely. Hence, acute telomere attrition and chromosomal end-to-end fusions occur frequently in AT ^-/-^ cells, which consistently supports our data.

Thus, this study substantiates the role of ATM in telomere maintenance. The lack of ATM shows genome instability in the form of telomere shortening, telomere shortening leading to fusion, complex translocations, MN and DNA damage when subjected to exogenous damage. Supporting our hypothesis, the abovementioned statements suggest that ATM has an essential role in telomere repair in addition to its activities upon DNA damage. Besides, cells from AT ^-/-^ patients are hypersensitive and susceptible to DNA damage caused by cytotoxic chemical, physical, and biological agents. Hence, the study elucidates the detrimental clastogenic effects of arsenic and genomic insults induced by ionizing radiation on normal human lung fibroblasts and ATM compromised cells *in* *hetero* and homozygous states. Post-treatment, AT ^-/-^ cells exhibit accelerated telomere shortening compared to AT ^+/-^ and normal cells, making them susceptible to genomic instability and abnormal cellular proliferation.

## Conclusion

ATM is crucial for the maintenance of genome homeostasis with respect to DNA damage. As this protein is crucial to both dividing and differentiated cells, its absence is only detrimental to the stability of the genome. ^[ 2]^ Telomeres are constitutive structures for maintaining the stability of human chromosomes. ^[ 12]^ It is suggested that AT patients are at a great risk even after exposure to low doses of arsenite and ionizing radiation. As ATM is involved in telomere repair, the lack of ATM heightens abrogation of telomeres as end replication does not occur. ^[ 51]^ Chromosome end-to-end fusions and chromosome instability are known to be critical initiators of carcinogenesis. However, the exact molecular mechanism interactions of telomere associated group of proteins and ATM complex is not well known. ATM heterozygosity combined with occupational or environmental exposures to harmful background radiations can synergistically trigger genomic instability as a function of chromosomal aberrations in individuals. Early interventions with the help of cytogenetic and molecular profiling of ATM, can benefit patients helping to manage the associated clinical conditions worldwide.

Apart from classical research and diagnostic-based applications, screening for genome stability finds its utility in space. Space tourism is a newly introduced luxury that will potentially become a reality in the near future. Space explorations contemporarily demand a stable and healthy genome as humans are no longer in their own niche and environment. Different environmental factors, such as vacuum, solar UV radiation, charged particles, ionizing radiation, surface charging, and temperature extremes may become detrimental with the background of genomic instability. ^[ 52]^ Hence, conventional cytogenetic profiling for understanding the genetic signatures for the health of our genome, continues to be powerful in this current era of modern technology and advancements.
